# *CYP3A4 *and *CYP3A5 *genotyping by Pyrosequencing

**DOI:** 10.1186/1471-2350-6-19

**Published:** 2005-05-09

**Authors:** Adam A Garsa, Howard L McLeod, Sharon Marsh

**Affiliations:** 1 Washington University School of Medicine, Department of Medicine, Division of Oncology, St. Louis, MO 63110, USA; 2 The Siteman Cancer Center, St. Louis, MO 63110, USA

## Abstract

**Background:**

Human cytochrome P450 3A enzymes, particularly CYP3A4 and CYP3A5, play an important role in drug metabolism. CYP3A expression exhibits substantial interindividual variation, much of which may result from genetic variation. This study describes Pyrosequencing assays for key SNPs in *CYP3A4 *(CYP3A4*1B, CYP3A4*2, and CYP3A4*3) and *CYP3A5 *(CYP3A5*3C and CYP3A5*6).

**Methods:**

Genotyping of 95 healthy European and 95 healthy African volunteers was performed using Pyrosequencing. Linkage disequilibrium, haplotype inference, Hardy-Weinberg equilibrium, and tag SNPs were also determined for these samples.

**Results:**

CYP3A4*1B allele frequencies were 4% in Europeans and 82% in Africans. The CYP3A4*2 allele was found in neither population sample. CYP3A4*3 had an allele frequency of 2% in Europeans and 0% in Africans. The frequency of CYP3A5*3C was 94% in Europeans and 12% in Africans. No CYP3A5*6 variants were found in the European samples, but this allele had a frequency of 16% in the African samples. Allele frequencies and haplotypes show interethnic variation, highlighting the need to analyze clinically relevant SNPs and haplotypes in a variety of ethnic groups.

**Conclusion:**

Pyrosequencing is a versatile technique that could improve the efficiency of SNP analysis for pharmacogenomic research with the ultimate goal of pre-screening patients for individual therapy selection.

## Background

The human cytochrome P450 3A (CYP3A) subfamily of enzymes plays an important role in drug metabolism. The four CYP3A genes lie within a 218 kb region of chromosome 7q22.1 in the following order: *CYP3A5*, *CYP3A7*, *CYP3A4*, and *CYP3A43*. CYP3A enzymes, primarily CYP3A4 and CYP3A5, catalyze the metabolism of a multitude of exogenous and endogenous compounds. As the most abundant group of CYPs in the liver and small intestine, CYP3A enzymes strongly affect the oral bioavailability and clearance of many drugs, and it is estimated that CYP3A enzymes are involved in the metabolism of over half of the drugs currently approved by the Food and Drug Administration [1-4].

Interindividual variation in CYP3A expression is substantial. Protein expression in liver and small intestine varies up to 40-fold, leading to variation in drug metabolism [2,5]. Genetic variation within the *CYP3A *genes may contribute to interindividual variability in drug metabolism. It has been suggested that approximately 90% of inter-individual differences in hepatic CYP3A activity are due to genetic variation [6]. Single nucleotide polymorphisms (SNPs) are the most common form of genetic variation in the CYP3A genes.

CYP3A4*1B is a 5' untranslated region -392A>G transition in *CYP3A4 *[7]. A number of associations between CYP3A4*1B and clinical phenotypes have been found. Rebbeck et al. have shown that prostate cancer patients are more likely to have the CYP3A4*1B allele than healthy controls, and this has been confirmed in other studies [7-9]. Additionally, homozygous wild-type (CYP3A4*1A/*1A) individuals have an increased risk for developing leukemia after epipodophyllotoxin therapy [10].

Relatively little is known about the effects of the other commonly studied *CYP3A4 *SNPs, CYP3A4*2 and CYP3A4*3. CYP3A4*2 is a SNP in exon 7 (15713T>C) that results in a Ser222Pro change. *In vitro *kinetic studies have shown that CYP3A4*2 has a 6-to 9-fold reduced intrinsic clearance for nifedipine compared to wild-type [11]. CYP3A4*3 is a 1334T>C transition causing a Met445Thr change. Although this SNP occurs within a conserved region, no difference in testosterone, progesterone, or 7-benzyloxy-4(trifluoromethyl)coumarin metabolism was found [12].

CYP3A5*3C is an IVS3-237A>G (6986A>G) transition within intron 3 of *CYP3A5 *[13]. This transition creates an alternative splice site in the pre-mRNA, leading to the production of aberrant mRNA with a premature stop codon [13]. This SNP leads to polymorphic expression of CYP3A5. CYP3A5*3C homozygotes lack CYP3A5 expression, while individuals with at least one CYP3A5*1 wild-type allele express CYP3A5 [13]. Polymorphic expression of CYP3A5 may account for some of the interindividual variation in clearance of CYP3A substrates. Indeed, *CYP3A5 *genotype is predictive of tacrolimus doses for lung and kidney transplant recipients [14,15].

CYP3A5*6 is a 14690G>A synonymous mutation that causes the formation of a splice variant mRNA. Exon 7 is deleted, resulting in a frameshift and a truncated protein [13]. Very little is known about the effects of this SNP, although CYP3A5*6 was found to have no effect on midazolam clearance in a small sample size [16].

For further analysis of these SNPs and their relations to clinical outcomes, an accurate, rapid, and cost efficient method of SNP evaluation is needed. This study describes the use of Pyrosequencing to assay key *CYP3A4 *and *CYP3A5 *SNPs.

## Methods

### Genotyping

PCR was performed on DNA from 95 healthy European volunteers and 95 healthy African volunteers, after IRB approval and written informed consent [17,18]. PCR primers were designed using Primer Express Version 2.0 (ABI, Foster City, CA, USA) and Pyrosequencing Primer SNP Design Version 1.01 software [19]. Primer sequences and PCR conditions are described in Table 1. PCR was carried out using 1–5 ng genomic DNA, 0.6 nmol each of forward and reverse oligonucleotide PCR primers (one of which is biotinylated) (Integrated DNA Technologies, Coralville, Iowa, USA) and 1X AmpliTaq Gold PCR Master Mix (Applied Biosystems, CA, USA), containing 255U (0.05 U/ml) AmpliTaq Gold DNA polymerase, Gene Amp PCR Gold Buffer (30 mmol/L Tris-HCL, 100 mmol/L KCl, pH 8.05), 400 mM dNTP and 5 mmol/L MgCl_2_. Pyrosequencing was carried out as described [20] using internal primer diluted in 1X Annealing Buffer (20 mmol/L Tris-Acetate, 2 mmol/L MgAc_2_), 2X Binding Wash Buffer II pH 7.6 (10 mmol/L Tris-HCL, 2M NaCl, 1 mmol/L EDTA, 0.1% Tween20), Streptavidin Sepharose Beads (Amersham Biosciences, Uppsala, Sweden), 0.2 M NaOH,70% Ethanol, and a PSQ HS96 SNP reagent kit (Pyrosequencing AB, Uppsala, Sweden). Samples were analyzed on a PSQ HS96A instrument with pyrosequencing software (Biotage, Uppsala, Sweden). A Tecan pipetting robot (Tecan, Research Triangle Park, NC, USA) was used for all of the steps apart from the addition and transfer of the sepharose beads.

### Statistics

Pairwise linkage disequilibrium (|D'|), haplotype inference, and Hardy-Weinberg equilibrium were determined using the Polymorphism and Haplotype Analysis Suite [21,22]. Tag SNPs were determined using SNPtagger [23,24].

## Results and discussion

Genotyping data from the European and African samples are shown in Table 2. All results are in Hardy-Weinberg equilibrium. CYP3A4*1B allele frequencies were 4% for Europeans and 82% for Africans. No CYP3A4*2 alleles were found in either the European or African population samples. CYP3A4*3 had an allele frequency of 2% in Europeans and 0% in Africans. The frequency of CYP3A5*3C was 94% in Europeans and 12% in Africans. No CYP3A5*6 variants were found in the European samples, but this allele had a frequency of 16% in the African samples. There were no individuals homozygous for both CYP3A5*3C and CYP3A5*6 in either population.

The *CYP3A4 *and *CYP3A5 *genes lie in close proximity (136 kb) to one another on chromosome 7q22.1, so haplotypes were determined across both genes for each population (Table 3). In Europeans, haplotype 1 was the predominant haplotype, with a 90% frequency. Haplotypes 2, 3, and 5 were also observed, with frequencies of 5.5%, 1.8%, and 1%, respectively. Haplotype 4 was not observed, but it has an inferred frequency of 1.8%. The African population had five observed haplotypes: Haplotype 4 (57%), Haplotype 2 (15%), Haplotype 5 (12%), Haplotype 6 (11%), and Haplotype 7 (3%). Haplotype 1 was not observed, but it has an inferred frequency of 0.6%. No loci are significantly linked in either population (data not shown). In addition, no haplotype tag SNPs could be identified in either population. However, genotyping Europeans for CYP3A5*3C could be used to identify the haplotype of 95.5% of the population. Similarly, genotyping Africans for CYP3A4*1B and CYP3A5*3C could be used to identify the haplotype of 84% of the population.

The frequency of CYP3A4*1B in Europeans (4%) is consistent with other studies [25]. The CYP3A4*1B frequency in Africans (82%) is much higher than in Europeans, and it is also higher than the 35–67% frequency seen in African Americans [25]. The rare CYP3A4*2 allele was not found in either of our population samples. To date CYP3A4*2 has only been described in a Finnish Caucasian population, with an allele frequency of 2.7% [11]. The CYP3A4*3 allele frequency in Europeans (2%) is consistent with frequencies reported in other studies [25].

CYP3A5*3C frequency shows dramatic interethnic variation. In Europeans, the CYP3A5*3C variant is the predominant allele (94% frequency), but this allele has a much lower frequency in the African population (12%). CYP3A5*6 frequency also shows interethnic variation. The CYP3A5*6 allele was not found in Europeans, but it was found in the African population at a frequency of 16%. Other studies have also failed to find CYP3A5*6 in Europeans, but this has been found in African Americans at a frequency of 13–16% [16,26].

Haplotype also shows interethnic variation. Haplotype 1 is the predominant haplotype in Europeans, with haplotypes 2–5 occurring at low frequencies. In contrast, haplotype 4 is the most common haplotype in Africans, and haplotypes 2, 5, and 6 all occur at frequencies greater than 10%. However, the presence of homozygous variants was rare for *CYP3A4 *in Europeans and for *CYP3A5 *in Africans. Consequently, the limits of *in silico *determining haplotype frequencies in these populations should be taken into account. Larger population studies are necessary for a more accurate understanding of CYP3A4-CYP3A5 haplotypes. Interethnic variation highlights the need to analyze clinically relevant SNPs and haplotypes in a variety of ethnic groups. An understanding of the genetic variation that exists in various populations will aid in tailoring health care to different populations.

## Conclusion

Restriction fragment length polymorphism (RFLP) is the predominant method of SNP analysis used to assay *CYP3A4 *and *CYP3A5 *SNPs in previous studies [14,27]. Pyrosequencing offers several advantages over RFLP. For RFLP analysis, a SNP must alter a restriction enzyme cutting site. This limitation precludes many SNPs from RFLP analysis. Pyrosequencing assays can be designed for the vast majority of SNPs, making it a versatile alternative. Pyrosequencing also requires less time than RFLP. Post-PCR, Pyrosequencing steps take approximately 30 minutes for 96 samples, whereas enzyme digestion (1–2 h) and gel electrophoresis for RFLP take significantly longer. Additionally, Pyrosequencing assays are readily transferable to any lab with the appropriate equipment and require no on-site optimization. This procedure could improve the efficiency of SNP analysis for pharmacogenomic research with the ultimate goal of pre-screening patients for individual therapy selection.

## Competing interests

The author(s) declare that they have no competing interests.

## Authors' contributions

AG performed the experimental analysis and drafted the manuscript. AAG, HLM and SM interpreted the results. HLM and SM conceived of the study design and implementation and helped draft the manuscript. All authors read and approved the final manuscript.

## Pre-publication history

The pre-publication history for this paper can be accessed here:



## Figures and Tables

**Table 1:** Primer sequence, PCR and Pyrosequencing conditions for *CYP3A4 *and *CYP3A5 *SNP analysis.

SNP	Forward Primer^*a*^(5'-3')	Reverse Primer^*a *^(5'-3')	Number of Cycles	Annealing Temperature (° C)	Internal Primer^*bc*^(5'-3')	Sequence to Analyze^*d*^
CYP3A4*1B	aggacagcccatagagacaagg	*atcaatgttactggggagtcc	55	55	F-ccatagagacaagggca	A/GGAGA
CYP3A4*2	aacaatccacaagacccctt	*atcttcaaatgtactacaaatcactga	55	55	F-tttggatccattctttc	TCTC/TAAT
CYP3A4*3	cgtggaaccagattcagcaa	*gaaggagaagttctgaaggactctg	55	65	F-ccagaaactgcattgg	CAT/CGAGG
CYP3A5*3C	*cccacgtatgtaccacccagc	attagggtgtgacacagcaaga	55	65	R-ccaaacagggaagaga	TAC/TTG^e^
CYP3A5*6	*tctttggggcctacagcatg	aaagaaataatagcccacatacttattgagag	55	62	R-agaaaccaaattttaggaa	CTTC/TTTAG^e^

^a^* = biotin molecule attached

^*b*^F = forward primer

^*c*^R = reverse primer

^*d *^Simplex entry nucleotide information for Pyrosequencing

^e^Assays on reverse complement strand

**Table 2:** Genotype and allele frequencies for *CYP3A4 *and *CYP3A5 *SNPs in European and African populations. Figures in brackets are 95% confidence intervals.

SNP	European						African					
	n^*a*^	Wild Type	Heterozygous	Variant	p	q	n^*a*^	Wild Type	Heterozygous	Variant	p	q

CYP3A4*1B	93	86	7	0	0.96 (0.92–0.98)	0.04 (0.02–0.08)	88	3	25	60	0.18 (0.11–0.27)	0.82 (0.73–0.89)
CYP3A4*2	56	56	0	0	1	0	87	87	0	0	1	0
CYP3A4*3	94	90	4	0	0.98 (0.95–0.99)	0.02 (0.01–0.05)	93	93	0	0	1	0
CYP3A5*3C	95	0	12	83	0.06 (0.03–0.10)	0.94 (0.90–0.97)	95	72	23	0	0.88 (0.83–0.92)	0.12 (0.08–0.17)
CYP3A5*6	92	92	0	0	1	0	91	64	25	2	0.84 (0.78–0.89)	0.16 (0.11–0.22)

^*a *^n = number of passed samples out of 95 samples tested

**Table 3:** Haplotype analyses for *CYP3A4 *and *CYP3A5*.

	CYP3A4*1B	CYP3A4*2	CYP3A4*3	CYP3A5*3C	CYP3A5*6	*% European*	*% African*
Hap1	A	C	T	G	G	90	0.6^*a*^
Hap2	A	C	T	A	G	5.5	15
Hap3	A	C	C	G	G	1.8	0
Hap4	G	C	T	A	G	1.8^*a*^	57
Hap5	G	C	T	G	G	1	12
Hap6	G	C	T	A	A	0	11
Hap7	A	C	T	A	A	0	3

^*a *^inferred frequency
